# Supplementary material for: Translation in amino-acid-poor environments is limited by tRNAGln charging
Source: eLife. 2020 Dec 8;9:e62307. doi: 10.7554/eLife.62307 (PMC7744096; doi:10.7554/eLife.62307)
Supplement: Supplementary file 1. — Sequences of oligonucleotides used for tRNA charging assay (Figures 1 and 2) and for Northern blotting (Figure 1G) are listed. [file elife-62307-supp1.docx]

**Supplementary File 1.** Additional oligonucleotide sequences (not listed in the key resources table). Sequences of oligonucleotides used for tRNA charging assay (Figure 1 and 2) and for Northern blotting (Figure 1G) are listed.

| **Name** | **Assay** | **Sequence** |
| --- | --- | --- |
| ValMAC_FW | qPCR primer,  tRNA charging assay | 5’-GTTTCCGTAGTGTAGTGGTTATCACGTTCG-3’ |
| ValMAC_RV | qPCR primer,  tRNA charging assay | 5’-GAGAATTCCATGGTGTTTCCGCCC-3’ |
| iMetCAT_FW | qPCR primer,  tRNA charging assay | 5’-AGCAGAGTGGCGCAGCG-3’ |
| iMetCAT_RV | qPCR primer,  tRNA charging assay | 5’-GAGAATTCCATGGTAGCAGAGGATGGTTTCG-3’ |
| eMetCAT_FW | qPCR primer,  tRNA charging assay | 5’-GCCTCSTTAGCGCAGTAGGTAG-3’ |
| eMetCAT_RV | qPCR primer,  tRNA charging assay | 5’-GAGAATTCCATGGTGCCCCSTS-3’ |
| GlnCTG_FW | qPCR primer,  tRNA charging assay | 5’-GGTTCCATGGTGTAATGGTNAGCACTCTG-3’ |
| GlnCTG_RV | qPCR primer,  tRNA charging assay | 5’-GAGAATTCCATGGAGGTTCCACCGAGATTTG-3’ |
| LeuWAG_FW | qPCR primer,  tRNA charging assay | 5’-GGTAGYGTGGCCGAGCG-3’ |
| LeuWAG_RV | qPCR primer,  tRNA charging assay | 5’-GAGAATTCCATGGCAGYGGTGGG-3’ |
| ArgACG_FW | qPCR primer,  tRNA charging assay | 5’-GGGCCAGTGGCGCAATG-3’ |
| ArgACG_RV | qPCR primer,  tRNA charging assay | 5’-GAGAATTCCATGGCGAGCCAGC-3’ |
| yPhe_FW | qPCR primer,  tRNA charging assay | 5’-GCGGAYTTAGCTCAGTTGGGAGAG-3’ |
| yPhe_RV | qPCR primer,  tRNA charging assay | 5’-GAGAATTCCATGGTGCGAAYTCTGTGG-3’ |
| tRNA^GlnCTG^ | Northern probe | 5’- /5Biosg/CTAACCATTACACCATGGAAC-3’ |
| tRNA^ValMAC^ | Northern probe | 5’-/5Biosg/GATAACCACTACACTACGGAA-3’ |
| tRNA^iMetCAT^ | Northern probe | 5’-/5Biosg/GCTTCCGCTGCGCCACTCTGC-3’ |
